# Re‐Evaluation of Rat Corneal Damage by Short‐Wavelength UV Revealed Extremely Less Hazardous Property of Far‐UV‐C^†^

**DOI:** 10.1111/php.13419

**Published:** 2021-05-03

**Authors:** Sachiko Kaidzu, Kazunobu Sugihara, Masahiro Sasaki, Aiko Nishiaki, Hiroyuki Ohashi, Tatsushi Igarashi, Masaki Tanito

**Affiliations:** ^1^ Department of Ophthalmology Faculty of Medicine Shimane University Izumo Japan; ^2^ Ushio Inc. Tokyo Japan

## Abstract

Corneal damage‐induced various wavelength UV (311, 254, 235, 222 and 207 nm) was evaluated in rats. For 207 and 222‐UV‐C, the threshold radiant exposure was between 10 000 and 15 000 mJ cm^−2^ at 207 nm and between 3500 and 5000 mJ cm^−2^ at 222 nm. Penetrate depth to the cornea indicated by cyclobutene pyrimidine dimer (CPD) localization immediately after irradiation was dependent on the wavelength. 311 and 254 nm UV penetrate to corneal endothelium, 235 nm UVC to the intermediate part of corneal stroma, 222 and 207 nm UVC only to the most outer layer of corneal epithelium. CPD observed in corneal epithelium irradiated by 222 nm UVC disappeared until 12 h after. The minimum dose to induce corneal damage of short‐wavelength UV‐C was considerably higher than the threshold limit value (TLV^®^) promulgated by American Conference of Governmental Industrial Hygienists (ACGIH). The property that explains why UV‐C radiation at 207 and 222 nm is extremely less hazardous than longer UV wavelengths is the fact that this radiation only penetrates to the outermost layer of the corneal epithelium. These cells typically peel off within 24 h during the physiological turnover cycle. Hence, short‐wavelength UV‐C might be less hazardous to the cornea than previously considered until today.

## Graphical abstract

Comparison of the lowest observed adverse effects level (LOAEL), the no observed adverse effect level (NOAEL) in this study and the threshold limit value (TLV^®^) regulated by The American Conference of Governmental Industrial Hygienists (ACGIH). Radiant exposure threshold is estimated to be between LOAEL and NOAEL; however, there is a large difference from TLV^®^ at the far UV‐C band, such as 207 nm and 222 nm. Our results suggested that the TLV^®^ of the far UV‐C band may be much higher than previously thought.

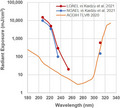

## INTRODUCTION

Ultraviolet (UV) radiation from sunlight is divided into three spectral regions: UV‐A (wavelengths, 315–400 nm), UV‐B (wavelengths, 280–315 nm) and UV‐C (wavelengths, 200–280 nm). Because UV‐C is almost completely absorbed by the atmospheric ozone layer and cannot reach to the earth’s surface (1), the influence of UV‐C on the cornea has not been sufficiently studied. However, human exposure to UV‐C is possible from welding arcs and artificial sources, such as fluorescent lamps, mercury‐vapor lamps, light‐emitting diodes, other lamps used to sterilize or control infection (2).

It has been generally thought that all UV can be harmful to the human body. In particular, the 254‐nm UV‐C, primary emission line of the conventional germicidal lamps (mercury lamps), is particularly harmful because it is near the maximum absorption wavelength of nucleic acids (260–275 nm), and therefore widely used for disinfection (3). However, recently it came to be known that far‐UV‐C, short‐wavelength UV‐C, such as 207 or 222 nm, would be safer for the human body while it had a disinfection effect since these wavelengths penetrate only to the stratum corneum, the outermost layer of the skin comprised of dead cells (4, 5, 6). In a previous study, we reported that 222‐nm UV‐C did not induce detectable corneal damage in rats, whereas 254‐nm UV‐C induced corneal damage even from a slight exposure dose (7). Furthermore, after the repetitive irradiation of 222‐nm UV‐C for to detect long‐term, delayed abnormalities of the eye and skin, none were observed in *xeroderma pigmentosum* complementation group A (*Xpa)*‐knockout mice (8). Irradiation at 222 nm appeared to be less hazardous to the cornea, as well as the skin, and this fact indicates that far‐UV‐C can be used in whole‐room germicidal applications even with persons present—unlike the more stringent precautions required for 254‐nm UV‐C disinfection.

UV‐C at 222 nm will become one of the important tools for prevention of infectious diseases such as COVID‐19 because very low doses of 222‐nm UV‐C efficiently inactivate human coronaviruses (9, 10). At present, the threshold limit value (TLV^®^) at 222 nm recommended by the American Conference of Governmental Industrial Hygienists (ACGIH) is 22 mJ cm^−2^ per day (11). However, this value was determined based upon the threshold studies performed about 50 years ago (12, 13, 14). Considering the expected use of shorter wavelength UV‐C as UV germicidal irradiation (UVGI) in future, it will be all the more important to reassess the threshold corneal damage by UV and reconsider the TLV^®^. Therefore, after initially focusing on 222 nm UV‐C, we expanded our investigations to evaluate the wavelength dependence of the rat corneal damage by UV‐B and UV‐C radiation. In this study, we revealed extremely less hazardous property of far‐UVC, short‐wavelength 207‐nm and 222‐nm UV‐C. Far‐UVC appears to be less hazardous to the cornea than had been previously considered and indicates that higher dose irradiation should be permissible.

## MATERIALS AND METHODS

### Animals

All procedures were performed according to the Association for Research in Vision and Ophthalmology Statement for the Use of Animals in Ophthalmic and Vision Research and The Shimane University Guidelines for Animals in Research (IZ29‐76, IZ31‐47, IZ29‐96). Five‐week‐old male Sprague‐Dawley rats were obtained from Charles River Laboratories Japan Inc. (Kanagawa, Japan) and maintained in our colony room for 7 to 10 days before the experiments. Twelve‐week‐old male BALB/c mice were obtained from Japan SLC Inc. (Shizuoka, Japan), and 10‐week‐old Japan white rabbit (2.0–2.3 kg body weight) was obtained from Biotek Co., Ltd (Saga, Japan). The light intensity in the cages was 10 to 20 lux. All animals were kept in a 12‐h (7 AM to 7 PM) light–dark cycle at 23°C.

Porcine eyes were obtained from slaughterhouse on a day to perform experiments and stored at 4°C until UV irradiation. Since the eyes were obtained after enucleation, the strain, sex, age and left or right side of the eye were unknown.

### Estimation of the duration of light exposure

**Figure 1:**
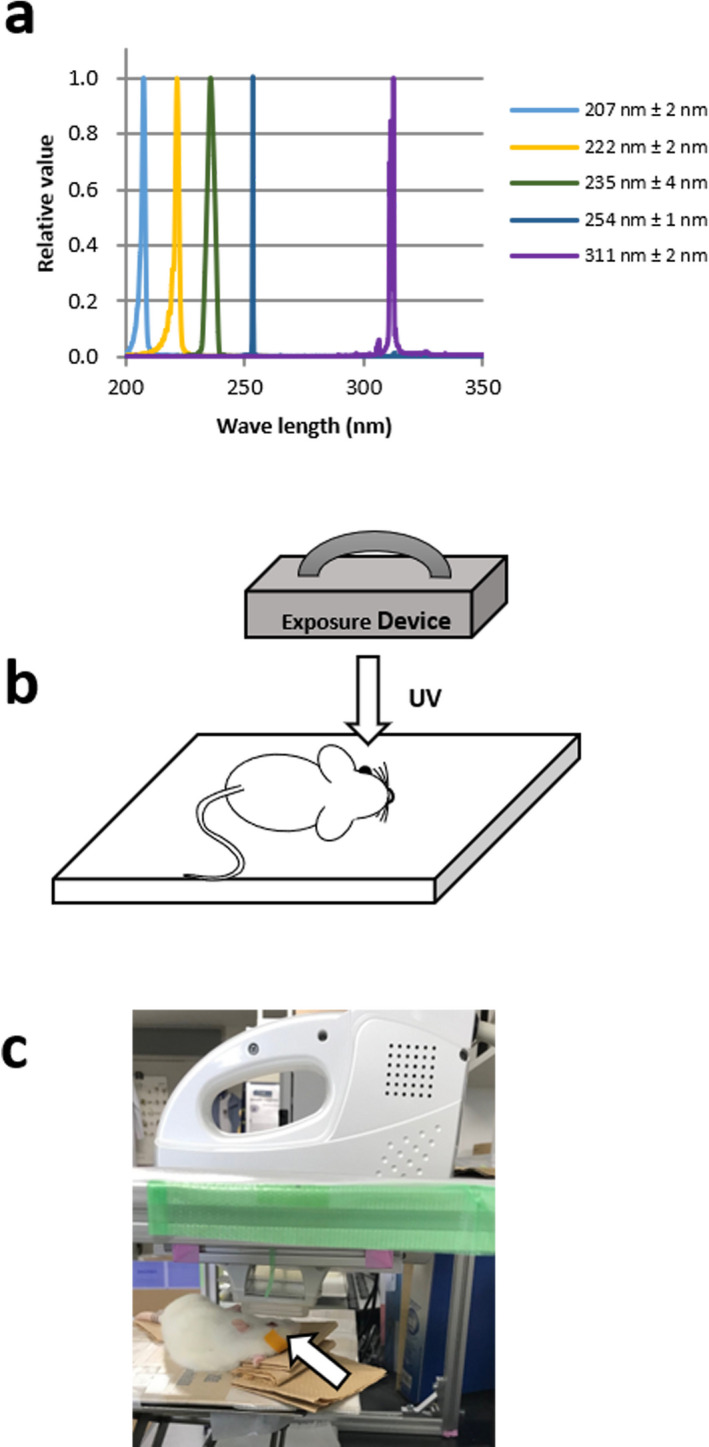
(a) Spectral distributions of UV light exposed to rats. (b)(c) UV light was irradiated to the cornea at a right angle. The counter eye was closed by tape (arrow) to prevent drying.

Before the start of each exposure, the irradiance was measured at the cornea with a UV meters. The exposure duration was determined by dividing the target corneal radiant exposure, by the measured irradiance. Rats were exposed to UV radiant energy with peaks at 207, 222, 235, 254 and 311 nm (Fig. 1a). The details of the UV meter, UV irradiation device and irradiation condition were shown in Table 1.

**Table 1:** Constitution of the irradiation device and irradiation condition.

Peak Wavelength (nm)	Light source	Type	Filter/Spector meter	Wave guide	Irradiated area (mm)	Irradiation power (mW cm^−2^)	Radiant Exposure (mJ cm^−2^)	Exposure duration (seconds)	FWHM (nm)	UV meter
207	KrBr Excimer lamp	Ushio Inc. SafeZoneUV‐C device	Band path (200–225 nm)	Quadrangular pyramid shape	16 × 18	0.83	30	231	2	USHIOS‐172/UIT250
							150	1154		
							600	3333		
							1000	4000		
							1500	6000–8333		
							2500	10 000		
							10 000	10 968–11 568		
							15 000	15 776–16 116		
222	Kr‐Cl Excimer lamp	Ushio Inc. SafeZoneUV‐C device	Band path (200–230nm)	No	30 × 32	4.2	30	6	2	USHIOS‐172/UIT250
							150	30		
							600	120–143		
							1500	300–357		
							2500	595		
							3500	833		
							5000	1190		
235	Xenon short arc lamp with internal mirror	Ushio Inc. UXR‐300UV	Refractive Band path filter (235 ± 5 nm) and Shimadzu SPG120UV spectrometer	No	2 × 2.25	0.077	10	90	4	OceanOptics QE‐pro Spectrometer
							30	182–380		
							50	450–620		
							100	606–1240		
							300	1818‐2700		
254	Low pressure mercury lamp	As one SUV‐4	No	No	47 × 73	1.3	20	18	1	USHIO UVD‐S254
							30	27		
							50	45		
							100	91		
							300	273		
311	Narrow band UV‐B lamp	PHILIPS TL20W/01RS	No	No	39 × 55	2.3	30	13–15	2	Ushio UVD‐S313
							150	65–75		
							600	261–300		

### UV exposure

For rats, mice and rabbits, irradiation was performed under anesthesia, and for porcine, irradiation was performed to the cornea removed after death. Before exposure, the rats were housed in dark boxes. After anesthesia was induced by an intramuscular injection of a mixture of ketamine (120 mg kg^−1^) and xylazine (6 mg kg^−1^), the pupils were dilated 0.5% tropicamide and 0.5% phenylephrine hydrochloride eye drops (Santen Pharmaceuticals Co., Ltd., Osaka, Japan) as described previously (7, 15). The center of the cornea was exposed to the UV light delivered at a right angle (Fig. 1b,c). During exposure, diluted saline (×2) was instilled onto the corneal surface to prevent drying. The body of the rat was covered with a black sheet except for the eye to prevent any UV skin damage. The details of exposure device and irradiation conditions were summarized in Table 1. After exposure, the rats were housed in cyclic light (10–20 lux, 12‐h light–dark cycle) until the eye enucleation for each experiment.

UV radiation exposures of the mice and rabbits were performed as had been described for the rat exposures. Anesthesia was induced by an intraperitoneal injection of a mixture of ketamine (120 mg kg^−1^) and xylazine (6 mg kg^−1^) for mice and by an intramuscular injection of a mixture of medetomidine (0.15 mg kg^−1^) and midazolam (1.0 mg kg^−1^) and butorphanol tartrate (5.0 mg kg^−1^) for rabbits. The pupils were dilated and the cornea was exposed to the UV doses as mentioned above. Porcine corneas were dissected from the eyeball and immersed in cold saline until irradiation. The eyes were exposed to UV irradiation to the doses at the wavelengths specified above.

### Evaluation of corneal damage

**Figure 2:**
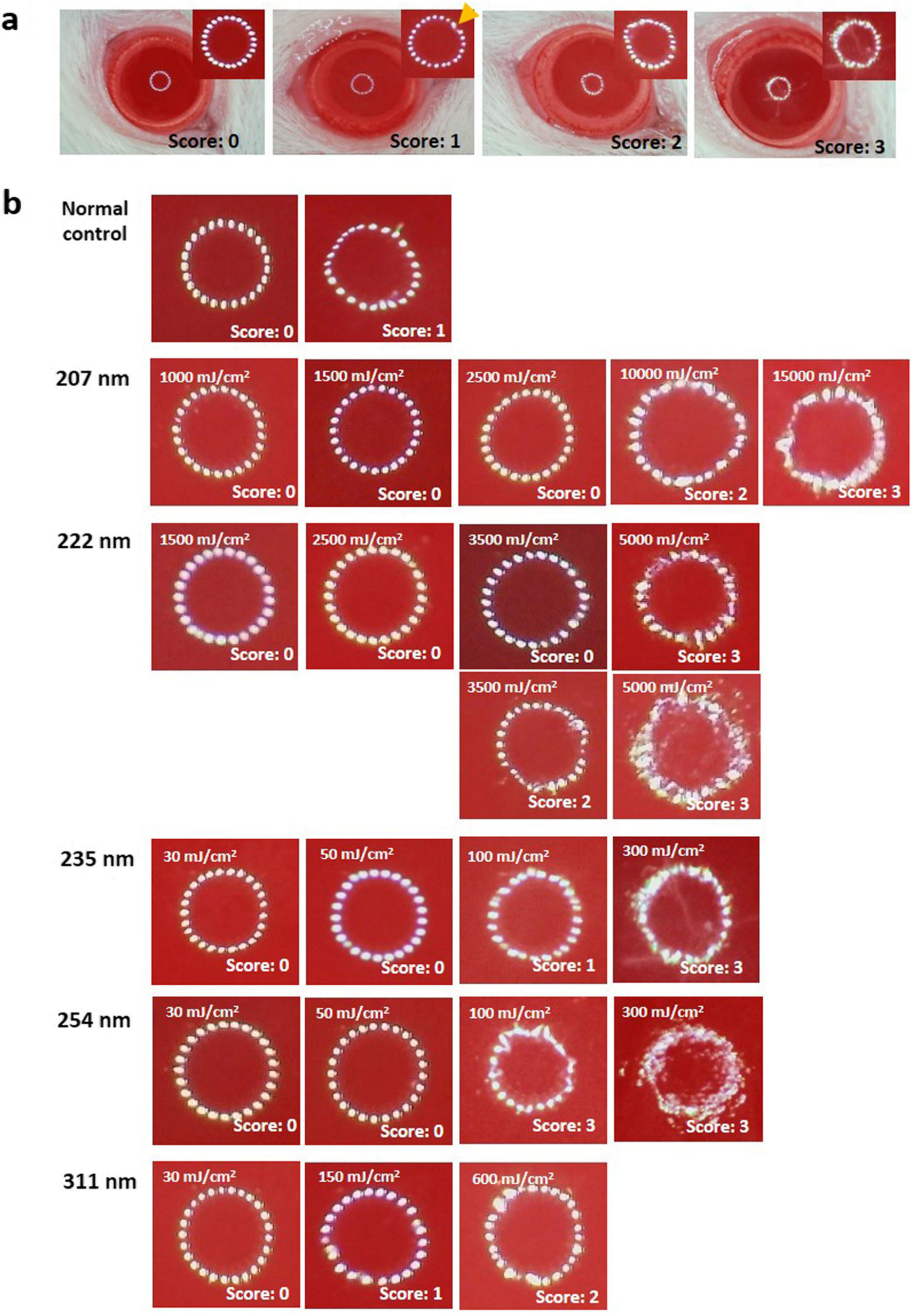
Scoring of mire ring irregularity. (a) Corneal surface damage was evaluated by mire ring according to the level irregularity. No damage; score 0, slightly irregular (arrow); score 1, irregular less than 50% circle; score 2, and irregular more than 50% circle; score 3. (b) Representative cases of mire ring in each wavelength and radiant exposure.

**Figure 3:**
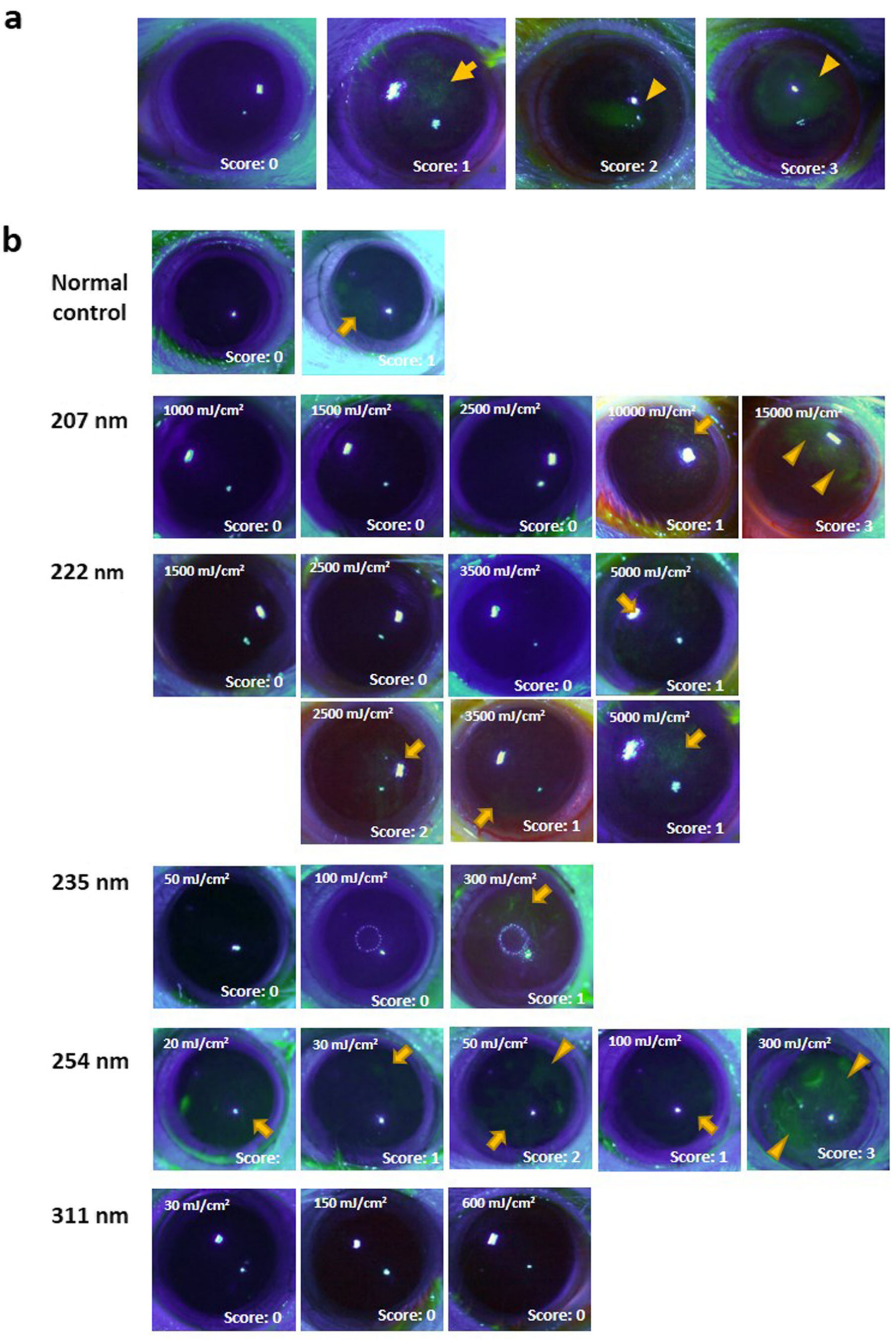
Scoring of Fluorescein staining. (a) Corneal epithelial defects detected by fluorescein staining were scored according to the erosion area. No damage; score 0, superficial punctate keratitis; score 1, erosion area less than 50% of cornea; score 2 and erosion area more than 50% of cornea; score 3. (b) Representative case of fluorescein stain in each wavelength and radiant exposure. Arrows indicate superficial punctate keratitis and arrowheads indicate erosion.

Twenty‐four hours after UV irradiation, we evaluated the rat corneal damage by the mire image of the cornea, a ring‐shaped light source (15), and fluorescein staining as described previously (7). The digitized color images of the rat corneas were obtained with a stereomicroscope (SMZ745T; Nikon, Tokyo, Japan) equipped with a digital Wi‐Fi camera (HIS; Kenis, Osaka, Japan). Corneal surface irregularities caused by UV radiation, which is thought to reflect corneal surface integrity, were scored according to the level of irregularity: no damage (score 0), slightly irregular (score 1), irregular less than 50% circle (score 2) and irregular more than 50% circle (score 3) (Fig. 2a). As described previously (7), corneal epithelial defects detected by fluorescein staining were also obtained and scored according to the erosion area using the following scale with slightly modification: no damage (score 0), superficial punctate keratitis (score 1), erosion area less than 50% of the entire cornea (score 2) and erosion area more than 50% of the entire cornea (score 3) (Fig. 3a). One masked researcher (KS) assessed the damage using a digitized color image.

### Hematoxylin–eosin staining

After euthanasia by an overdose of anesthesia and then cervical dislocation for rats and mice, or by CO^2^ gas for rabbits, both eyes were enucleated and fixed in 4% paraformaldehyde containing 20% isopropanol, 2% trichloroacetic acid and 2% zinc chloride for 24 h at room temperature. After alcohol dehydration, the eyes were embedded in paraffin, and 4‐μm‐thick sagittal sections were cut that contained the entire retina including the optic disk. The sections were stained with hematoxylin–eosin (H&E) to observe histological damage.

### DNA damage

We observed cyclobutane pyrimidine dimer (CPD) localization as a DNA damage marker. The sections were deparaffinized, and endogenous peroxidase activity was inactivated with 3% hydrogen peroxide for 10 min. We used a VECTASTAIN^®^ ABC mouse IgG kit (Vector Laboratories Inc., Burlingame CA) for immunostaining. After blocking with horse normal serum for 30 min at room temperature, the sections were incubated with anti‐cyclobutane pyrimidine dimer (CPD) (Cosmo Bio Co., Ltd., Tokyo, Japan) (1:400) antibody diluted with antibody diluent (Dako North America, Inc., Carpenteria, CA) for 1 h at 37°C and then with the anti‐mouse IgG biotinylated antibody for 30 min at 37°C. For the negative control experiments, the sections were incubated with antibody diluent without a primary antibody for 1 h at 37°C. After washing, the sections were incubated with ABC complex for 30 min at 37°C. Signals were developed with 3′,3′‐diaminobenzidine (ImmPACT ^TM^ DAB Peroxidase substrate kit, Vector Laboratories Inc.) in chromogen solution.

### Invasion depth of UV to the cornea

We used CPD localization in the cornea for the index of invasion depth of UV, because CPD was produced by UV reached to DNA. To compare the invasive depth of each UV wavelength, immediately after UV irradiation both eyes were enucleated, then stained as mentioned above. Radiant exposure in each wavelength was 600 mJ cm^−2^. Furthermore, the 222‐nm and 254‐nm UV penetration depths were examined for each animal. The radiant exposure was 600 mJ cm^−2^ at 222 nm and 50 mJ cm^−2^ at 254 nm.

### Change of CPD localization

To assess the DNA damage recovery of corneal epithelium, we observed the change of CPD localization in the cornea irradiated at 222 and 254 nm UV‐C. Radiant exposures were made with 600 mJ cm^−2^ and 50 mJ cm^−2^ at each wavelength. Both eyes were enucleated immediately after (0 h), 0.5, 1, 2, 4, 6, 9 and 12 h after UV‐C irradiation, then stained as mentioned above.

### Statistical analysis

Statistical analyses were performed using JMP version 14.2 software (JMP Statistical Discovery, Cary, NC). Mire ring scores and fluorescein staining scores were compared between the UV irradiated cornea and unexposed cornea (normal control) groups using the unpaired *t*‐test. *P* < 0.05 was considered significant.

## RESULTS

Anesthetized rats were exposed to UV radiant energy with spectral peaks at 207, 222, 235, 254 and 311 nm with a range of radiant exposures. Twenty‐four hours after irradiation, we evaluated rat corneal threshold damage using the mire image of the cornea, a ring‐shaped light source according to the level irregularity (Fig 2), and by fluorescein staining according to the erosion area (Fig. 3). Mire ring irregularity more than 50% of the entire circle was observed in all cornea irradiated at 254‐nm with 100 and 300 mJ cm^−2^ radiant exposures (*P* < 0.001 vs the unexposed control) (Table 2). In the eyes radiated at 311‐nm in the UV‐B, a significant difference was observed in corneas irradiated at 600 mJ cm^−2^ (*P* < 0.01 vs unexposed controls). In the eyes irradiated at 235‐nm (UV‐C), significant differences were observed in corneas irradiated at 300 mJ cm^−2^ (*P* < 0.05 vs unexposed controls). On the other hand, in the eyes irradiated at 222 nm, a 5000 mJ cm^−2^ radiant exposure was necessary to observe a significant difference (*P* < 0.001 vs unexposed control). In the eyes irradiated at 207‐nm, there were no significant differences. By fluorescein staining, significant differences were observed in all exposed corneas, even in the corneas irradiated at the lowest radiant exposure (20 mJ cm^−2^, *P* < 0.01 vs unexposed control) (Table 3). In the eyes exposed to 235‐nm UV‐C, a significant difference was observed in the cornea irradiated at 300 mJ cm^−2^ (*P* < 0.05 vs unexposed control) as well as by the mire ring evaluation. However, significant differences were not observed in the eyes irradiated at 207‐, 222‐ and 313‐nm UV with this dose.

**Table 2:** Mire ring score

Radiant Exposure (mJ cm^−2^)																
Wavelength	0	10	20	30	50	100	150	300	600	1000	1500	2500	3500	5000	10 000	15 000
Normal control	0.33 ± 0.52 (*n* = 6)	‐	‐	‐	‐	‐	‐	‐	‐	‐	‐	‐	‐	‐	‐	‐
207 nm	‐	‐	‐	0.53 ± 0.64 (*n* = 15)	‐	‐	0.13 ± 0.35 (*n* = 15)	‐	0.27 ± 0.59 (*n* = 15)	0.25 ± 0.50 (*n* = 4)	0.50 ± 0.84 (*n* = 6)	0.00 ± 0.00 (*n* = 6)	‐	‐	1.17 ± 0.41 (*n* = 6)	3.00 ± 0.00**^†††^** (*n* = 6)
222 nm	‐	‐	‐	0.00 ± 0.00 (*n* = 6)	‐	‐	0.33 ± 0.52 (*n* = 6)	‐	0.00 ± 0.00 (*n* = 8)	‐	0.00 ± 0.00 (*n* = 6)	0.33 ± 0.82 (*n* = 6)	1.00 ± 1.26 (*n* = 6)	2.83 ± 0.41**^†††^** (*n* = 6)	‐	‐
235 nm	‐	0.75 ± 0.50 (*n* = 4)	‐	0.67 ± 1.03 (*n* = 6)	1.17 ± 1.33 (*n* = 6)	1.00 ± 1.55 (*n* = 6)	‐	1.83 ± 1.17 **^†^** (*n* = 6)	‐	‐	‐	‐	‐	‐	‐	‐
254 nm	‐	‐	0.83 ± 1.17 (*n* = 6)	0.33 ± 0.82 (*n* = 6)	0.43 ± 0.53 (*n* = 7)	3.00 ± 0.00**^†††^** (*n* = 6)	‐	3.00 ± 0.00**^†††^** (*n* = 6)	‐	‐	‐	‐	‐	‐	‐	‐
311 nm	‐	‐	‐	0.50 ± 0.84 (*n* = 6)	‐	‐	1.00 ± 0.89 (*n* = 6)	‐	1.83 ± 0.75**^††^** (*n* = 6)	‐	‐	‐	‐	‐	‐	‐

Mire ring score. Data are expressed as mean ± standard deviation. **^†^** *P* < 0.05, **^††^** *P* < 0.01, **^†††^** *P* < 0.001 VS. The normal control (irradiation) groups by unpaired *t‐*test.

**Table 3:** Fluorescein staining score.

Radiant Exposure (mJ cm^−2^)																
Wavelength	0	10	20	30	50	100	150	300	600	1000	1500	2500	3500	5000	10 000	15 000
Normal control	0.17 ± 0.41 (*n* = 6)	‐	‐	‐	‐	‐	‐	‐	‐	‐	‐	‐	‐	‐	‐	‐
207 nm	‐	‐	‐	0.13 ± 0.52 (*n* = 15)	‐	‐	0.00 ± 0.00 (*n* = 15)	‐	0.00 ± 0.00 (*n* = 15)	0.25 ± 0.50 (*n* = 4)	0.33 ± 0.52 (*n* = 6)	0.17 ± 0.41 (*n* = 6)	‐	‐	1.00 ± 0.89 (*n* = 6)	2.00 ± 0.89**^†^** (*n* = 6)
222 nm	‐	‐	‐	0.00 ± 0.00 (*n* = 6)	‐	‐	0.00 ± 0.00 (*n* = 6)	‐	0.50 ± 0.93 (*n* = 8)	‐	0.00 ± 0.00 (*n* = 6)	1.17 ± 0.98 (*n* = 6)	1.00 ± 1.26 (*n* = 6)	0.67 ± 0.82 (*n* = 6)	‐	‐
235 nm	‐	0.25 ± 0.50 (*n* = 4)	‐	0.00 ± 0.00 (*n* = 6)	0.33 ± 0.52 (*n* = 6)	0.67 ± 0.52 (*n* = 6)	‐	1.00 ± 1.10**^†^** (*n* = 6)	‐	‐	‐	‐	‐	‐	‐	‐
254 nm	‐	‐	1.67 ± 0.82**^††^** (*n* = 6)	1.00 ± 0.00**^†††^** (*n* = 6)	1.33 ± 0.82**^†^** (*n* = 6)	0.83 ± 0.41**^†††^ (** *n* = 6)	‐	2.83 ± 0.41**^†††^** (*n* = 6)	‐	‐	‐	‐	‐	‐	‐	‐
311 nm	‐	‐	‐	0.00 ± 0.00 (*n* = 6)	‐	‐	0.00 ± 0.00 (*n* = 6)	‐	0.17 ± 0.41 (*n* = 6)	‐	‐	‐	‐	‐	‐	‐

Data are expressed as mean ± standard deviation. **^†^** *P* < 0.05, **^††^** *P* < 0.01, **^†††^** *P* < 0.001 VS. Normal control (irradiation) groups by unpaired t‐test.

**Figure 4:**
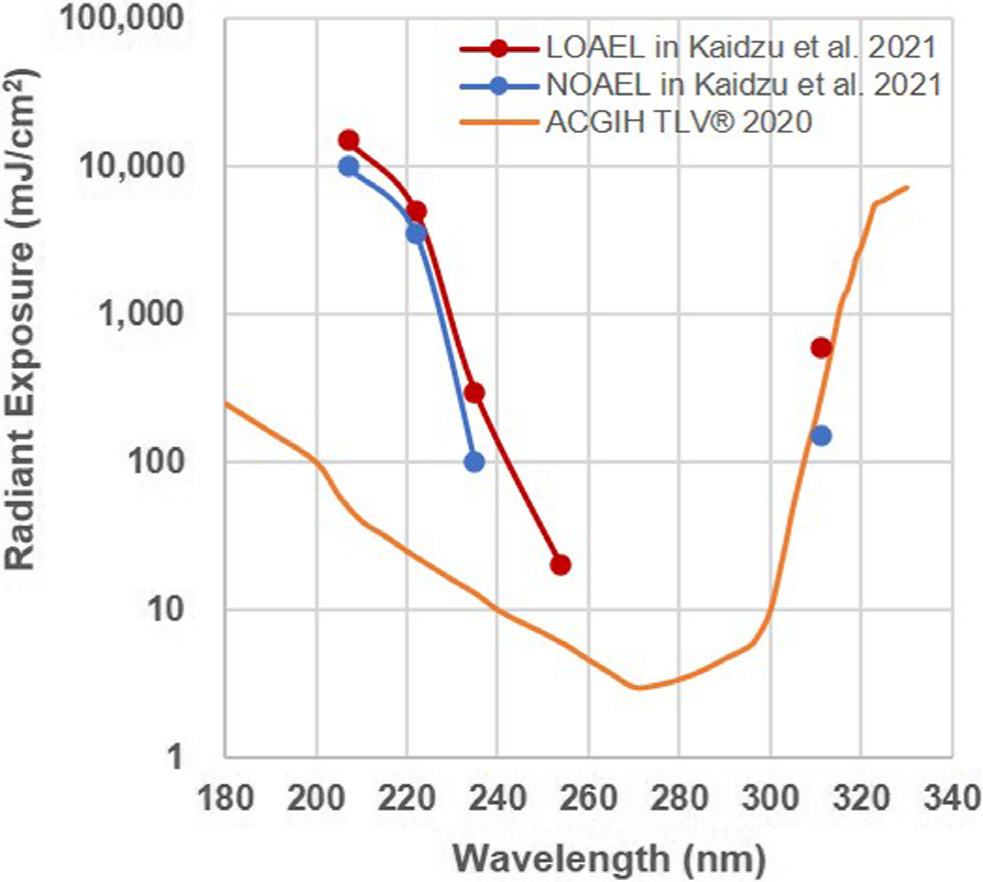
The lowest observed adverse effect level (LOAEL) and no observed adverse effect level (NOAEL) for corneal damage according to the mire ring evaluation and fluorescein staining score. The red line and dot show LOAEL, the blue line and dot show NOAEL, and the yellow line shows the threshold limit value (TLV^®^) published by American Conference of Governmental Industrial Hygienists (ACGIH) in 2020.

We assumed that the radiant exposure required for a significant difference seen in either the mire ring or fluorescein staining score was the lowest observed adverse effect level (LOAEL), and the radiant exposure to cause no corneal damage that is the highest of those tested in present study was the no observed adverse effect level (NOAEL) (Fig. 4). In the case that significant differences seen between the mire ring and fluorescein staining scores, we considered the lower radiant exposure to be the LOAEL at that wavelength. The most hazardous UV wavelength studied was 254 nm, which induced corneal damage at only 20 mJ cm^−2^ irradiation; that is, LOAEL at 254 nm was 20 mJ cm^−2^. The LOAEL at 235 nm was 300 mJ cm^−2^ and was 600 mJ cm^−2^ at 311 nm. The LOAEL radiant exposures at 207 nm and 222 nm to the cornea were 15 000 mJ cm^−2^ and 5000 mJ cm^−2^, respectively. The NOAEL at 235 nm was 100 mJ cm^−2^ and was 150 mJ cm^−2^ at 311 nm. The LOAEL radiant exposures at 207 nm and 222 nm to the cornea were 10 000 mJ cm^−2^ and 3500 mJ cm^−2^, respectively. For 254 nm, the NOAEL was unknown because corneal damage was observed at the lowest dose (20 mJ cm^−2^) in this study.

**Figure 5:**
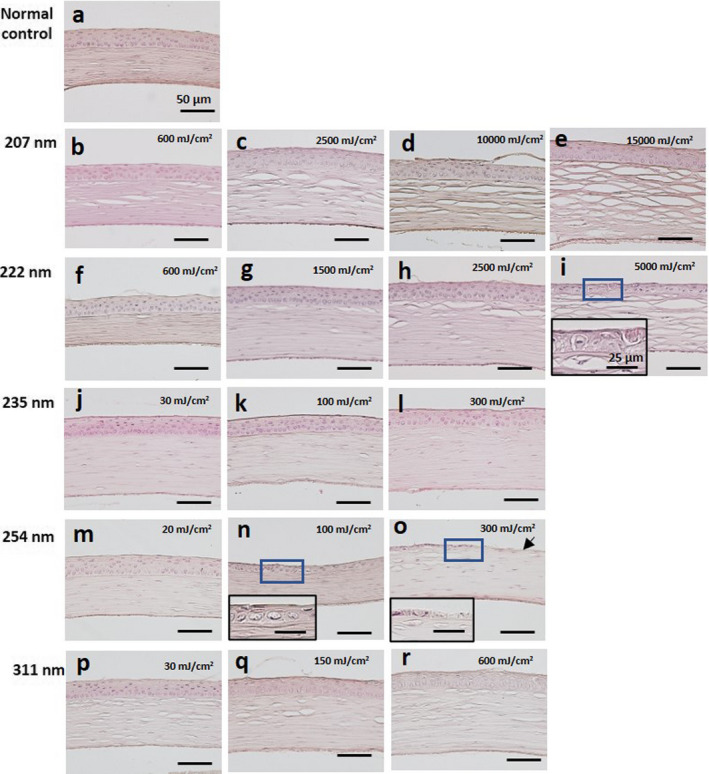
H&E staining of the cornea. (a) Unexposed normal control. (b–e) The cornea irradiated by 207‐nm UV‐C. (f–i) The cornea irradiated by 222‐nm UV‐C. (j–l) The cornea irradiated by 235‐nm UV‐C. (m–o) The cornea irradiated by 254‐nm UV‐C. (p–r) The cornea irradiated by 311‐nm UV‐B. The small box indicates the high‐magnification images of the part surrounded by the blue line. Arrow indicates exposed stroma. Bar = 50 µm in each panel and 25 µm in the high‐magnification images.

Histological damage was observed by hematoxylin–eosin (H&E) stain (Fig. 5). In a cornea irradiated at 254 nm with 100 mJ cm^−2^, the epithelium thinned. An extremely thin epithelium, consisting of one remaining layer of the stroma, is shown, and the stroma is partly exposed (arrow) in the cornea irradiated at 254‐nm with a dose of 300 mJ cm^−2^. In the cornea irradiated at 222‐nm with 5000 mJ cm^−2^, the epithelium became slightly thinner, although the cornea irradiated at less than 2500 mJ cm^−2^ appeared normal. The cornea irradiated at 207, 235‐nm and 311‐nm also appeared normal.

**Figure 6:**
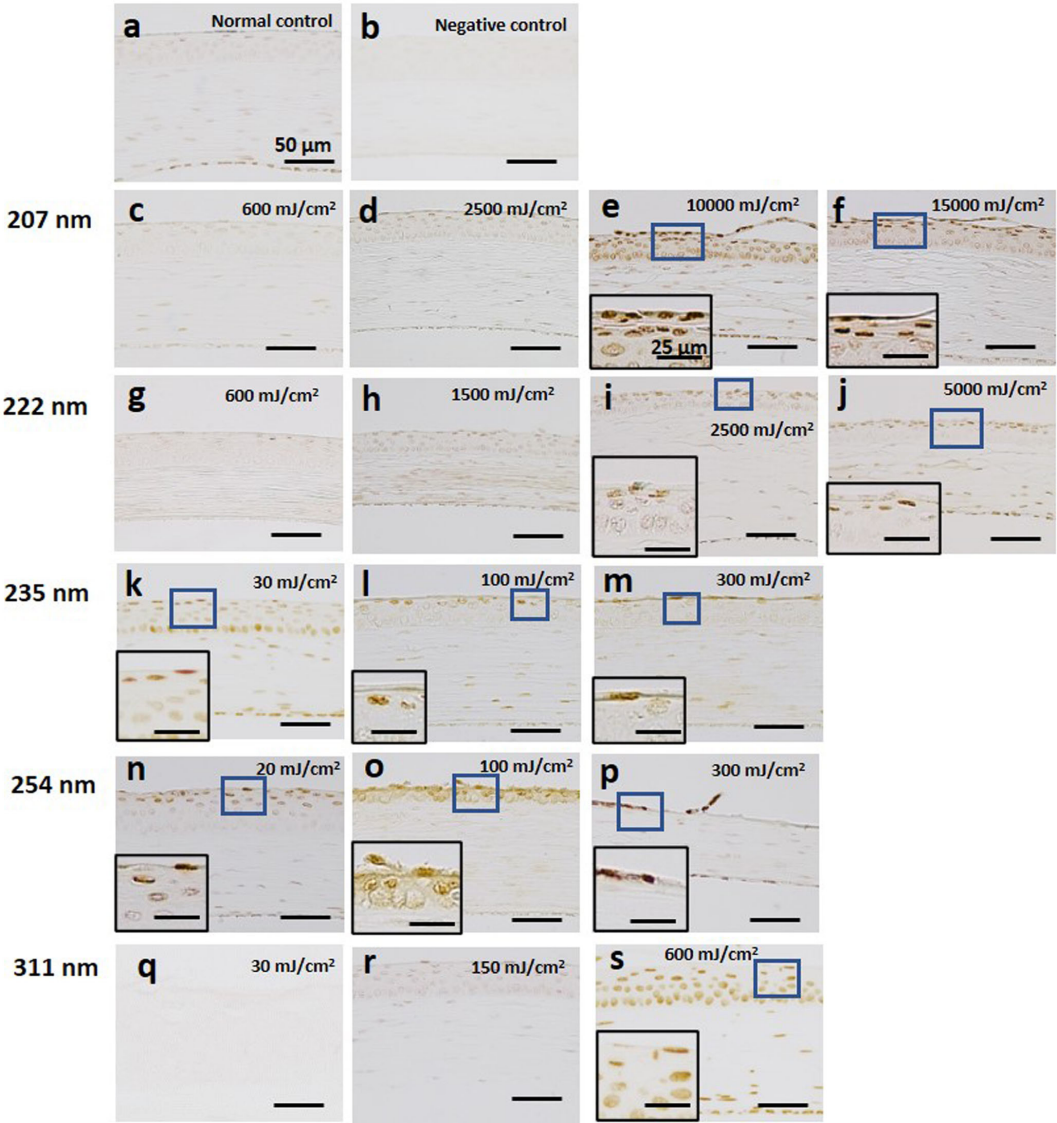
Expression of CPDs in the cornea. (a) Unexposed normal control. (b) Negative control. (c–f) The cornea irradiated by 207‐nm UV‐C. (g–j) The cornea irradiated by 222‐nm UV‐C. (k–m) The cornea irradiated by 235‐nm UV‐C. (n–p) The cornea irradiated by 254‐nm UV‐C. (q–s) The cornea irradiated by 311‐nm UV‐B. Small box indicates the high‐magnification images of the part surrounded by the blue line. Bar = 50 µm in each panel and 25 µm in high‐magnification images.

We used cyclobutane pyrimidine dimer (CPD) localization as a DNA damage marker (Fig. 6). Endothelial cells in the cornea without irradiation were slightly stained. In the cornea irradiated at 600 mJ cm^−2^ with 311‐nm (UV‐B), almost all cells existed in the corneal epithelium, stroma and endothelium were moderately stained although CPDs were not observed in the cornea irradiated less than 150 mJ cm^−2^. In the cornea irradiated at 20 mJ cm^−2^ at 254‐nm (UV‐C), most outer epithelial cells were strongly stained, and the cells existing near the stroma were not stained. In the cornea irradiated at 100 mJ cm^−2^, the thinned epithelium was entirely stained. In the cornea irradiated at 300 mJ cm^−2^, the remaining epithelial cells were strongly stained. On the other hand, in the cornea irradiated at 235‐nm (UV‐C), cells existing in one or two levels of the outermost layer of the corneal epithelium were stained, regardless of the level of radiant exposure. As for 222‐nm, CPDs were also observed in one or two levels of the outermost layer of the corneal epithelium irradiated to 2500 and 5000 mJ cm^−2^, and not in the corneas irradiated at less than 1500 mJ cm^−2^ radiant exposure. In the cornea irradiated at 207 nm, CPDs were observed in the 2–3 outermost layers when irradiated at more than 10 000 mJ cm^−2^, and not in the corneas irradiated at <2500 mJ cm^−2^.

**Figure 7:**
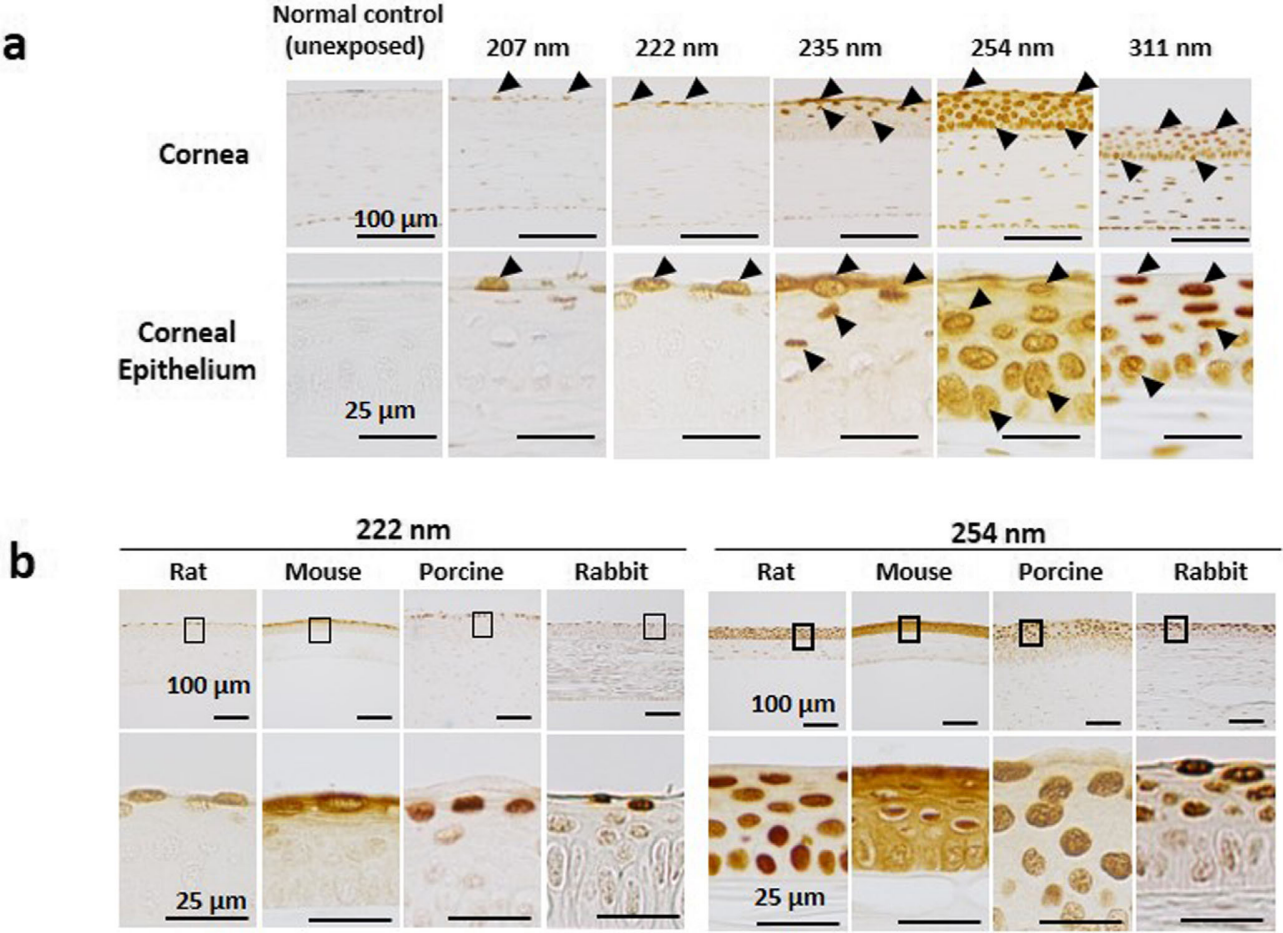
(a) CPD localization in the cornea immediately after UV irradiation. Arrows indicate PCD‐positive cells. Bar = 100 µm in the upper panel and 25 µm in the lower panel. (b) CPD localization in corneas of the various animals immediately after 222 nm or 254 nm in the UV‐C band. The part surrounded by a black line is indicated below with high magnification. Bar = 100 µm in the upper panel and 25 µm in the high‐magnification images.

In the cornea immediately after 313 or 254‐nm UV, CPDs were observed in all cells in the cornea, which indicated that these UV wavelengths penetrated to the corneal endothelial cells (Fig. 7a). CPDs were observed in cells of the middle layer of the corneal epithelium after irradiation by 235‐nm UV. CPDs were observed only in the outermost layer cells in the cornea irradiated by 222 and 207 nm UV‐C. We also observed CPD localization in corneas of the various animals immediately after 222 or 254 nm in the UV‐C band. Regardless of animal species and whether animal was alive (rat, mouse and rabbit) or dead (porcine) during irradiation, 222‐nm UV penetrated only the outermost layer of the corneal epithelium, whereas 254‐nm UV penetrated to the corneal endothelium (Fig. 7b).

**Figure 8:**
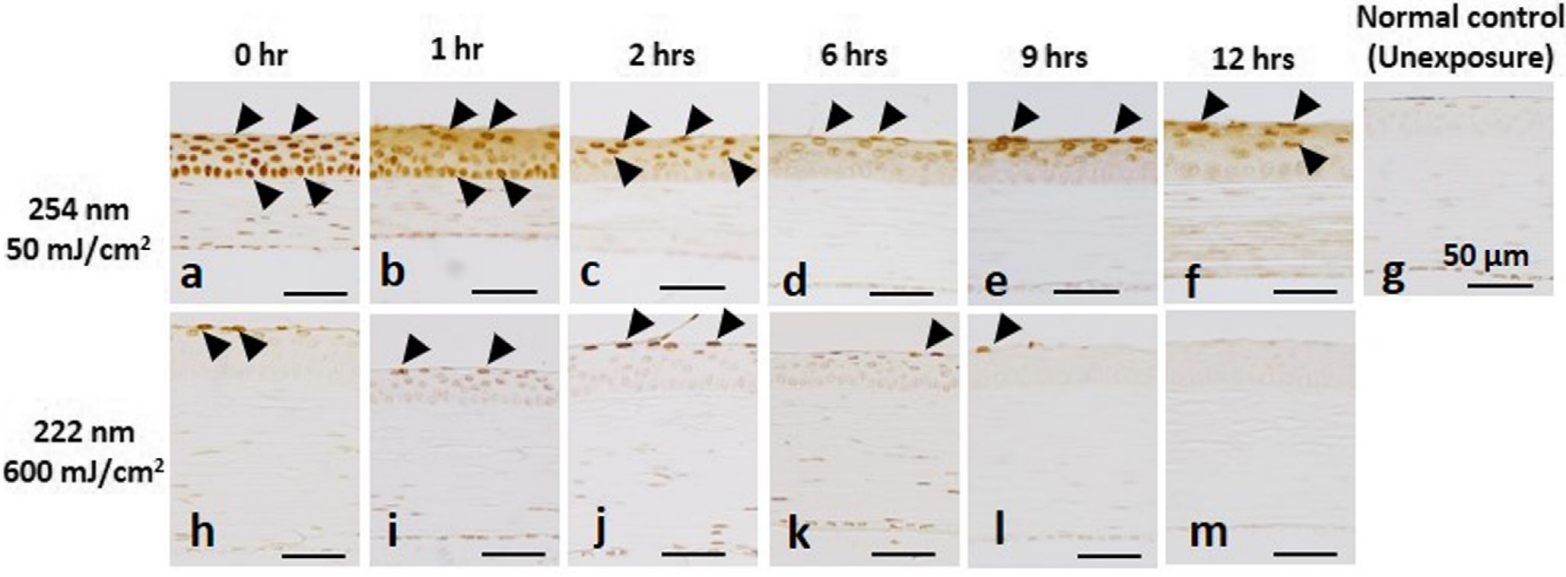
The change of CPD localization in the cornea when irradiated by 254‐nm (a–f) or 222‐nm UV‐C (h–m). (g) Negative control. Arrows indicate PCD‐positive cells. Bar = 50 µm in each panel.

We observed the change of CPD localization in the cornea when irradiated by 222‐nm or 254‐nm UV‐C (Fig. 8). In the cornea immediately after 254‐nm UV radiation, CPDs were observed in all corneal epithelial cell. CPDs gradually disappeared from the basal cells with increased time; however, they remained in the outermost layer even after 12 h. On the other hand, CPDs observed in the outermost outer layer of the cornea irradiated at 222‐nm also gradually disappeared and were no longer present after 12 h.

## DISCUSSION

Anesthetized rats were exposed to UV radiant energy with spectral peaks at 207, 222, 235, 254 and 311 nm with a range of radiant exposures. Twenty‐four hours after irradiation, we evaluated rat corneal threshold damage using the mire image of the cornea, a ring‐shaped light source, and by fluorescein staining. The most hazardous UV wavelength studied was 254 nm, which induced corneal damage at only 20 mJ cm^−2^ irradiation, that is, the LOAEL at 254 nm was 20 mJ cm^−2^. The LOAEL at 235 nm was 300 mJ cm^−2^ and was 600 mJ cm^−2^ at 311 nm. The LOAEL at 207 nm and 222 nm to the cornea was as follows: 15 000 mJ cm^−2^ and 5000 mJ cm^−2^, respectively, that is, 250 times and 750 times higher than the 254‐nm UV‐C LOAEL. On the other hand, the NOAELs at 235 nm and 311 nm were 100 and 150 mJ cm^−2^, and those at 207 nm and 222 nm were 10 000 and 3500 mJ cm^−2^, respectively. For 254 nm, the NOAEL was unknown because corneal damage was observed at the lowest dose (20 mJ cm^−2^) in this study; however, it is assumed to be less than 20 mJ cm^−2^. The threshold radiant exposure is lower than the LOAEL and higher than the NOAEL. Therefore, the threshold radiant exposure of 207 nm was estimated to be between 10 000 and 15 000 mJ cm^−2^; similarly those of 222 nm was between 3500 and 5000 mJ cm^−2^, 235 nm was between 100 and 300 mJ cm^−2^, 311 nm was between 150 and 600 mJ cm^−2^, and 254 nm is estimated to be <20 mJ cm^−2^. (Fig. 4).

These LOAEL values, particularly in the far‐UV‐C band – at 207 and 222 nm, differ from previous reports which had been the basis of the current ACGIH TLV^®^. Pitts and Tredici reported an LOAELs at 222‐nm for rabbit, primate and human of approximately 45, 20 and about 10 mJ cm^−2^, respectively (12, 14), but, by comparison, that for rats was 5000 mJ cm^−2^ in the present study. Most possible reasons might be the technical difference of irradiation to the animals. Broad‐spectrum xenon–mercury lamp used in the past studies had very low output near 222‐nm UV‐C and 10‐nm‐wide bandwidth, indicating stray light, which was more hazardous also irradiated the cornea that might have influenced the corneal damage evaluation. Furthermore, Chaney and Sliney (16) reviewed the hazard action spectrum where the action spectrum varied rapidly with a small change in wavelength and reported that large bandwidths caused significant plotting errors, requiring a change of the action spectrum. In the present study, we used a Kr‐Cl excimer lamp that produced a sharp spectral peak with a narrow half‐bandwidth of only 2 nm. Therefore, it is thought to have little impact on potential plotting or stray‐light errors. Of course, the lamp we used in the present study included longer and more hazardous UV wavelengths although this emission was extremely small. For the corneas irradiated with large UV radiant exposures, the influence of even trace amounts of the out‐of‐band, extra UV cannot be ignored because that radiant exposure became sufficient to induce corneal damage. We observed damage in the cornea irradiated at 222 nm with 5000 mJ cm^−2^ or at 207 nm with 15 000 mJ cm^−2^; however, it is very difficult to distinguish which UV induced the corneal damage, irradiated far‐UV‐C or extra stray UV. There is the possibility that the LOAEL of 222 nm or 207 nm UV‐C was therefore actually higher than estimated in present study. Another possibility for the discrepancy would be a difference in the criteria for corneal damage. Pitts et al. determined thresholds using epithelial haze, which was irregularity or crackled appearance of the corneal surface, and number of the debris in tear, which was thought to be sloughing of the outermost layer of corneal epithelium, etc. (12, 13, 14). On the other hand, we evaluated the corneal damage by mire ring, which should be almost equivalent to the criteria of epithelial haze and fluorescein staining, which has been used to diagnose corneal punctate keratitis in daily clinical care. We scored mire ring and fluorescein staining results, then assumed as cornel damage in the case that there was significant difference comparing with normal (unexposed) eye, although Pitts et al assumed any haze as an above threshold radiant exposure and represented the results as positive (+) or negative (−). The discrepancy in thresholds might be generated by a combination of these reasons mentioned above. In addition to our results, Sliney et al reported that radiant exposure of 193 nm UV‐C, a shorter wavelength than we studied to induce corneal damage in rabbit, lied between 1000 and 1500 mJ cm^−2^ (17). It will be certain that the threshold of far‐UV‐C is considerably higher comparing with UV‐C wavelengths near the maximum absorption range of nucleic acids. Certainly, ACGIH would determine a TLV^®^ after having considered all factors of safety. However, our results suggested that the TLV^®^ of the far‐UV‐C band, such as at 207 and 222 nm, may be much higher than previously thought.

In the skin, far‐UV‐C wavelengths penetrate only to the stratum corneum, the outermost layer of the skin comprised of dead cells (4, 5); therefore, far‐UV‐C cannot induce biological damage. On the other hand, the outermost layer of the corneal epithelium is comprised of living cells. The tear film above the corneal epithelium partly absorbs far‐UV‐C; however, over 80% of far‐UV‐C penetrates the tear film and reaches the outermost (wing‐cell) layer of the corneal epithelium (18), indicating that the tear film provides only very limited protection at these wavelengths. Then, why is far‐UV‐C less hazardous? It might be explained by the penetration depth in the cornea. The penetration depth of UV into the cornea depends approximately on the UV wavelength (Fig. 7a). In the cornea immediately after 313 nm UV‐B or 254 nm UV‐C irradiation, cyclobutene pyrimidine dimers (CPDs), the marker of UV damage of DNA, were observed in all cells in the cornea, indicating that these UV wavelengths penetrated to the corneal endothelial cells. CPDs were observed in cells of the middle layers of the corneal epithelium irradiated at 235 nm; however, the CPDs were observed only in the outermost layer cells in the cornea irradiated at 222 or 207 nm. CPDs, observed in the cornea immediately after 254‐nmUV irradiation, gradually disappeared from basal cells with advance of time; however, they remained in the outermost layer even after 12 h (Fig. 8). On the other hand, CPDs observed in the outermost layer in the corneas irradiated at 222 nm gradually disappeared and were no longer evident after 12 h. The corneal epithelium is comprised of 5–7 cell layers, with squamous cells in the outermost part, wing cells in the middle part and basal cells adjacent to the corneal stroma (19). The corneal epithelium is replaced within 5–7 days by basal cells gradually moving to the outer layer, and the squamous cell peering off, indicating that the outermost layer of the corneal epithelium is sloughed off within 24 h. Far‐UV‐C wavelengths, such as 207 and 222 nm, penetrate only to the outermost layer of the corneal epithelium and cells in these layers slough off in a normal cycle of physiological turnover. However, DNA repair, such as MutY DNA Glycosylase (Mutyh), 8‐Oxoguanine DNA Glycosylase (Ogg1) and human MutT Homolog 1 (MTH1) might be partly involved in the corneal protection against UV, the extremely low penetration into the cornea and the rapid turnover cycle of the corneal epithelium might be the primary reason of that far‐UV‐C is less hazardous.

It is reported that 222‐nm UVC shows sufficient inactivation of SARS‐CoV‐2 with small exposure doses (9, 10). As well as SARS‐CoV‐2, there will be possibility that an unknown virus appears in future. Far‐UV‐C, particularly 222 nm UV‐C, will become more important tools against any virus, because UV photons have an inactivation effect regardless of the virus mutation. At present, 222‐nm UV‐C is used at doses below the current ACGIH TLV^®^. However, if 222‐nm UV‐C could be available with higher exposure doses, its inactivation effects become higher and its application scene might become more widespread. It will be necessary to reconsider the TLV applicable to far‐UV‐C based on these current studies to permits a utilization of far‐UV‐C radiation more effectively; and hopefully, our results will play an important role in that updating process.
